# Comparative analysis of two timepoints on [^18^F]FAPI-42 PET/CT in various cancers

**DOI:** 10.1186/s41824-023-00186-1

**Published:** 2023-12-11

**Authors:** Xingyu Mu, Biyun Mo, Jie Qin, Zuguo Li, Weixia Chong, Yulong Zeng, Lu Lu, Lei Zhang, Wei Fu

**Affiliations:** Department of Nuclear Medicine, Affiliated Hospital of Guilin Medical University, No. 15 Lequn Road, Xiufeng District, Guilin, 541001 Guangxi Zhuang Autonomous Region China

**Keywords:** [^18^F]FAPI-42, 2-[^18^F]FDG, PET/CT, Two-timepoint

## Abstract

**Purpose:**

This study aimed to assess the biodistribution, detection rate, and uptake of the [^18^F]FAPI-42 at two distinct time intervals.

**Methods:**

This prospective study enrolled 60 consecutive patients (median age 59; range 35–74) referred to [^18^F]FAPI-42 PET/CT. [^18^F]FAPI-42 PET/CT was performed early and late timepoint after tracer injection for staging or restaging. Positive lesions specified for anatomic locations (primary or recurrent tumor, LN metastasis and other metastasis) by visual analysis at both timepoints. Semiquantitative analysis of the tracer activity in lesions as well as normal tissues at both time points were measured and compared. In a subgroup analysis, eleven patients underwent 2-[^18^F]FDG PET/CT within 1 week, the detection rate and uptake of lesion were compared between early [^18^F]FAPI-42 and 2-[^18^F]FDG.

**Results:**

Uptake of [^18^F]FAPI-42 in the late timepoint was significantly lower than the early timepoint in most organs (all *p* < 0.05), except for bone (SUV_mean_ 0.88 vs. 0.85; *p* = 0.218). Tracer retention at biliary system showed less frequent at early timepoint than late timepoint. A total of 194 lesions were detected in 60 patients. One lesion was only seen at early timepoint but not at late timepoint. Lesions on early [^18^F]FAPI-42 PET/CT had higher visual score than that of late image(23 vs. 6). The uptake of lesion decreased significantly from early to late timepoint (all *p* < 0.05). In subgroup analysis, early [^18^F]FAPI-42 illustrated higher detection rate, visual score, and uptake of lesion than that of 2-[^18^F]FDG PET/CT.

**Conclusion:**

Early [^18^F]FAPI-42 PET/CT provided consistent detection rates and lesion uptake, but less tracer retention in the biliary system compared to late images. Therefore, acquisition at early timepoint could be a feasible strategy for improving acquisition protocols of [^18^F]FAPI-42 PET/CT.

*Trial registration*: ChiCTR2200063441. Registered 28 September 2022—Retrospectively registered, https://www.chictr.org.cn/bin/project/edit?pid=149714.

**Supplementary Information:**

The online version contains supplementary material available at 10.1186/s41824-023-00186-1.

## Introduction

Fibroblast activation protein (FAP) is abundantly expressed in cancer-associated fibroblasts (CAFs) within the tumor microenvironment, while its presence in healthy tissues is minimal (Loktev et al. 2018). The introduction of FAP-specific small molecule inhibitors, known as FAPI, for positron emission tomography (PET) imaging marked a significant milestone (Kratochwil et al. 2019; Giesel et al. 2019). Numerous studies have since highlighted their rapid clearance from the body, outstanding diagnostic efficacy, and impressive tumor-to-background ratios (TBR) in detecting a variety of cancers (Wass et al. 2023; Yang et al. 2023; Hirmas et al. 2023). Given the observed attributes of FAPI PET, it shows potential in contributing to the determination of the TNM stage and could have implications for clinical decision-making in cancer treatments (Koerber et al. 2023). In some types of cancer, FAPI PET demonstrated even better tumor detection and staging, contrasting with 2-[^18^F]FDG on oncologic PET/computed tomography (CT) imaging (Yang et al. 2023).

Currently, molecular imaging probes targeting FAP predominantly utilize Gallium-68 (^68^ Ga) for PET imaging (Mori et al. 2023). Among these, ^68^[Ga]Ga-FAPI-04 and ^68^[Ga]Ga-FAPI-46 stand out as the most employed tracers, with exceptional diagnostic capabilities and notably straightforward workflow, such as no need for a complex fasting process, and short interval time from injection to acquisition (Ferdinandus et al. 2021). However, while these attributes underscore the benefits of ^68^[Ga]Ga-FAPI, its widespread clinical application is constrained by the short half-life of ^68^ Ga, elevated costs, and limited availability of radionuclides from the ^68^Ge/^68^ Ga generator (Mori et al. 2023).

The application of ^18^F-labeled FAPI compounds presents a compelling alternative, enhancing the performance limit in the clinical routine. Specifically, [^18^F]FAPI boasts benefits such as high production accommodating more examinations per batch, facilitating multicenter transportation, and delivering high-resolution imaging (Mori et al. 2023). Compared to ^68^[Ga]Ga-FAPI, [^18^F]FAPI exhibit similar characteristics, biodistribution and tracer kinetics, such as high affinity to tumor, swift renal clearance and minimal non-specific uptake in normal tissue (Hu et al. 2022). That made that [^18^F]FAPI has performed promising diagnostic performance for various cancers (Yang et al. 2023; Zhang et al. 2023; Watabe et al. 2023; Yao et al. 2022). However, a distinction between ^68^ Ga- and ^18^F-FAPI lies in the chelator (normally DOTA for ^68^[Ga]Ga-FAPI; NOTA for [^18^F]FAPI). This leads to increased lipophilicity in [^18^F]FAPI, resulting in excretion through the biliary system. Consequently, this may impact the visualization of gastrointestinal lesions and reduce its diagnostic efficacy (Wang et al. 2021).

In most of these studies, static PET acquisition was performed at an hour after the injection of [^18^F]FAPI (Yang et al. 2023; Qiao et al. 2023). While some research has identified 10–30 min as an optimal timepoint for ^68^[Ga]Ga-FAPI PET acquisition (Naeimi et al. 2023; Glatting et al. 2022), the ideal timepoint for [^18^F]FAPI PET remains unclear. Dynamic scans of [^18^F]FAPI-42 PET/CT from a prior study revealed that tumor activity peaked at 18 min and then began to decrease, with gallbladder activity increasing thereafter (Hu et al. 2022). In preclinical assessment, tumor presented highest uptake on early [^18^F]FAPI-42 PET/CT (Huang et al. 2023). These findings prompted us to investigate the variations between early and late timepoints for [^18^F]FAPI-42 PET/CT in cancer imaging.

In this study, we assessed the biodistribution, detection rate, and tumor uptake of [^18^F]FAPI-42 across various cancers between early and late for [^18^F]FAPI-42 PET/CT. Additionally, in a subset of patients, the detection rate and tumor uptake on early [^18^F]FAPI-42 PET/CT was compared to that of 2-[^18^F]FDG PET/CT.

## Methods and materials

### Study design and patients

This study received approval from the Ethics Committee of our hospital (Approval No. 2022WJWZCLL-01) and was registered in the Chinese Clinical Trial Registry (ChiCTR2200063441). Every participant provided written informed consent.

Between August 2022 and July 2023, 65 patients were consecutively enrolled from The Affiliated Hospital of Guilin Medical University. Patients were referred for one of three primary reasons:1. Determining lesion characteristics and disease severity 2. Restaging due to inconclusive findings on conventional imaging 3. Regular follow-up. Five patients were excluded in analysis due to patients with non-oncology disease. Finally, 60 patients were analyzed.

### Radiopharmaceuticals and PET/CT imaging

The Affiliated Hospital of Guilin Medical University's Department of Nuclear Medicine routinely synthesized 2-[^18^F]FDG using standardized procedures. The synthesis and labeling of [^18^F]AlF‑NOTA‑FAPI‑42 (abbreviated as [^18^F]FAPI-42) have been previously documented (Mu et al. 2023). Both 2-[^18^F]FDG and [^18^F]FAPI-42 maintained a radiochemical purity of over 95%.

In each patient, whole-body [^18^F]FAPI-42 PET was acquired twice: early (mean 22 min) and late (mean 73 min) after injection of radiotracer. Eleven patients underwent sequential 2-[^18^F]FDG and [^18^F]FAPI-42 PET/CT scans within a week. The 2-[^18^F]FDG PET/CT protocols adhered to the European Association of Nuclear Medicine's international guidelines (Boellaard et al. 2015). All images were captured using the Ingenuity TF PET/CT scanner (Philips, Amsterdam, Holland). A low-dose CT scan (tube voltage: 120 kV, tube current: 50 mAs, slice thickness: 5.0 mm, pitch: 1.0) was first taken for attenuation correction, followed by PET imaging (2.5 min per bed position, 6–7 PET bed positions).

### Image interpretation

All images were interpreted by Q.J. and Z.L. with more than 5 years of experience in PET interpretation. These specialists were unaware of the 2-[^18^F]FDG PET/CT outcomes when evaluating the [^18^F]FAPI-42 PET/CT. Image assessments encompassed both visual analysis and quantitative measurement. Consensus was reached after discussions. Elevated 2-[^18^F]FDG/[^18^F]FAPI-42 uptake, paired with anomalies in CT density or signals, was deemed positive after ruling out physiological uptake, trauma, infection, and inflammation. The number of lesions was recorded and categorized by region: primary tumor or recurrence, metastatic lymph nodes, as well as distant metastasis. A visual scoring system was developed to assess lesion detection capabilities. This system, detailed in previous studies (Liu et al. 2023; Qin et al. 2022), was based on three criteria: lesion area, count, and tracer uptake. The scoring system was applied to compare lesions detected by early [^18^F]FAPI-42 PET with those by late [^18^F]FAPI-42 PET and 2-[^18^F]FDG PET. The detail of this visual scoring system was depicted in Additional File 1.

Tracer uptake and biodistribution in normal organs were quantified using mean standardized uptake values (SUV_mean_)at both early and late timepoints. For the evaluation of these organs: Specific region of interest (ROI) measurements was employed. For smaller organs like the thyroid, parotid gland, and salivary glands, a spherical ROI with a 1 cm diameter was used. For larger organs such as the brain, muscle, liver, pancreas, spleen, kidney, aortic lumen content (blood pool), lung, and bone, a 2 cm diameter spherical ROI was adopted. The ROI was placed entirely within the organ parenchyma. The presence of biliary and intestinal tracer retention and was recorded for both late and early time-points. The tracer uptake of each lesion was assessed using multiple SUV metrics, including maximum standardized uptake values (SUV_max_), SUV_mean_, and peak standardized uptake values (SUV_peak_), at both early and late timepoints. For SUV calculations: Circular volumes of interest were manually outlined around tumor lesions on trans-axial slices at the late timepoint. These were then automatically transferred to images from the early timepoint using the MedEx software (MedEx Technology Limited Corporation, China), with a 3-dimensional ROI set at a 40% iso-contour. Image contrast was quantified using tumor-to-background ratios (TBR). The formula employed the ratio of SUV_max_ of the lesion to the SUV_mean_ of background tissue, which included measurements from the blood pool (TBR_blood_) and liver (TBR_liver_) tissue at both early and late timepoints, respectively.

### Statistics

Statistical evaluations were conducted using IBM SPSS Statistics version 26.0 and GraphPad Prism version 9.0. Continuous variables are depicted as [median (IQR)], while categorical variables are shown as [n, (%)]. Chi-square tests were used to compare presence of biliary and intestinal tracer retention between two timepoints. The Wilcoxon matched-pairs signed-rank test was employed to compare tracer uptake in normal organs and lesions between two timepoints as well as that in 2-[^18^F]FDG. All tests were two-sided, with *p* < 0.05 denoting statistical significance.

## Results

Between August 2022 and July 2023, 65 patients were consecutively enrolled in this study. Five were excluded due to benign diseases, resulting in 60 patients for the analysis. Of these, 25 patients underwent [^18^F]FAPI-42 PET/CT for primary staging, while 35 were assessed using [^18^F]FAPI-42 PET/CT for restaging. Eleven patients underwent 2-[^18^F]FDG within 1 week. Baseline patient characteristics are provided in Table 1.

**Table 1:** Patient demographics

Characteristic	N (%)
No. of patients	60
Gender	
Male	43 (71.7%)
Female	17 (28.3%)
Median age (y)	59 (Range, 35–74)
Indication of PET/CT	
Staging	25 (41.7%)
Restaging	35 (58.3%)
Time of image acquisition	
Early timepoint(min)	22(IQR 18,25)
Late timepoint(min)	73(IQR 64,80)
Interval time between two acquisitions(min)	51(IQR 43,60)
Tumor type	
Head and neck tumor	3 (5.0%)
Thyroid cancer	5 (8.3%)
Lung cancer	15 (25.0%)
Breast cancer	2 (3.3%)
Gastrointestinal cancer	23 (38.3%)
Cervical cancer	2 (3.3%)
Tumor of urinary system and male genital organs	10 (16.8%)

### Biodistribution in normal organs

**Fig. 1:**
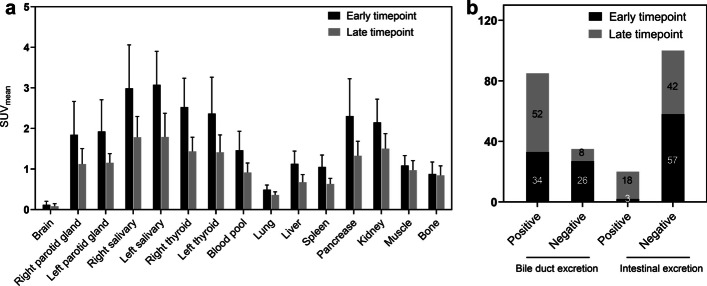
**a** SUV_mean_ of normal organs at early and late timepoint in patients with cancer on [^18^F]FAPI-42 PET/CT. **b** The tracer retention of biliary and intestinal between early and late timepoint on [^18^F]FAPI-42 PET/CT. Vertical bar with error bar shows the mean with standard error

The biodistribution of normal organs at the early and late [^18^F]FAPI-42 PET/CT is depicted in Fig. 1a. Uptake of [^18^F]FAPI-42 in the late timepoint was significantly lower than the early timepoint in most organs (14 out of 15 organs, all *p* < 0.05), except for bone (SUV_mean_ 0.88 vs. 0.85; *p* = 0.218; Additional file 1: Table S1). In early vs. late imaging, tracer retention in biliary was presented in 34/60 (56.7%) vs. 52/60 (86.7%) patients respectively (*p* < 0.001), and intestinal 3/60(5%) vs. 18/60(30%) patients, respectively (*p* < 0.001) (Fig. 1b).

### Visual and quantitative evaluation

**Fig. 2:**
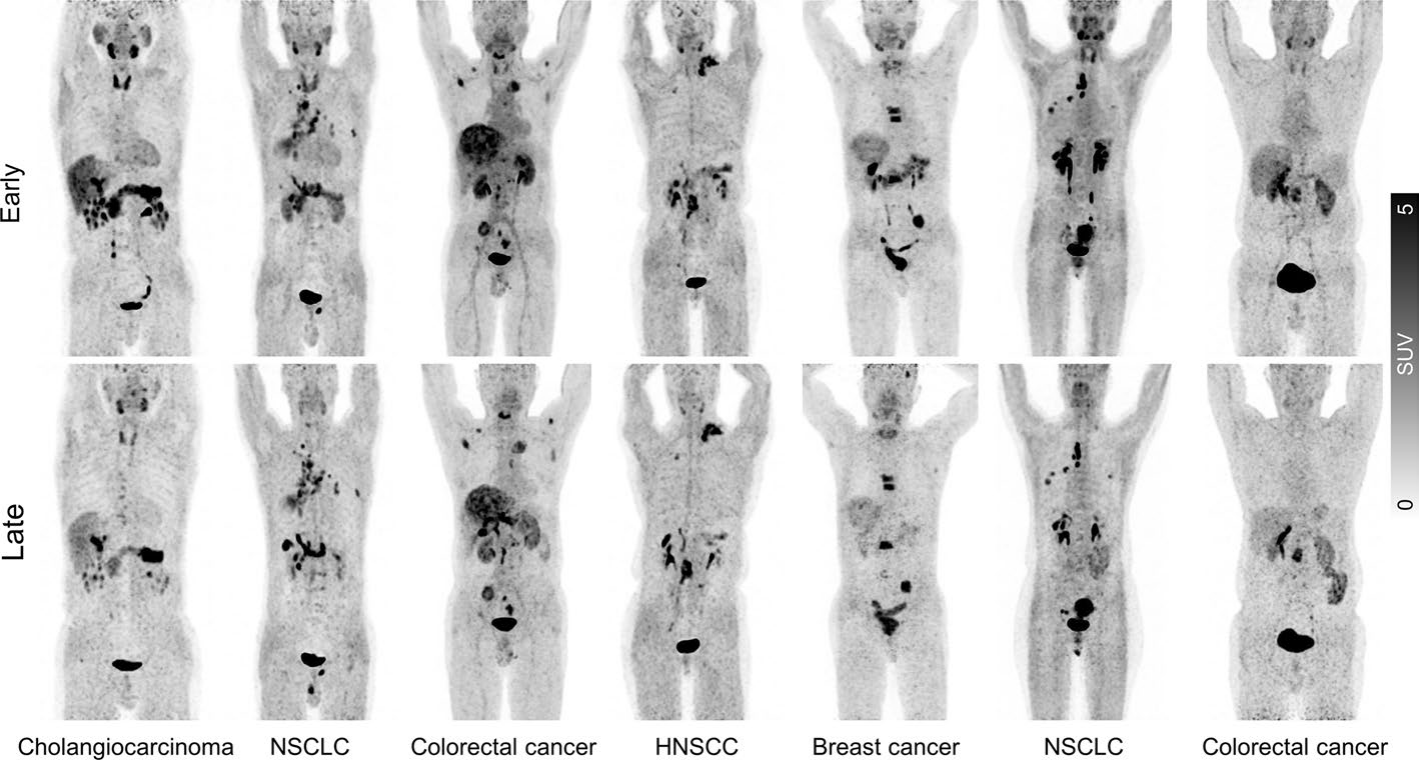
Maximum-intensity-projection images of [^18^F]FAPI-42 PET/CT at early and late timepoint in different types of cancer. NSCLC = Non-small cell lung cancer; HNSCC = Head and neck squamous cell carcinoma

**Fig. 3:**
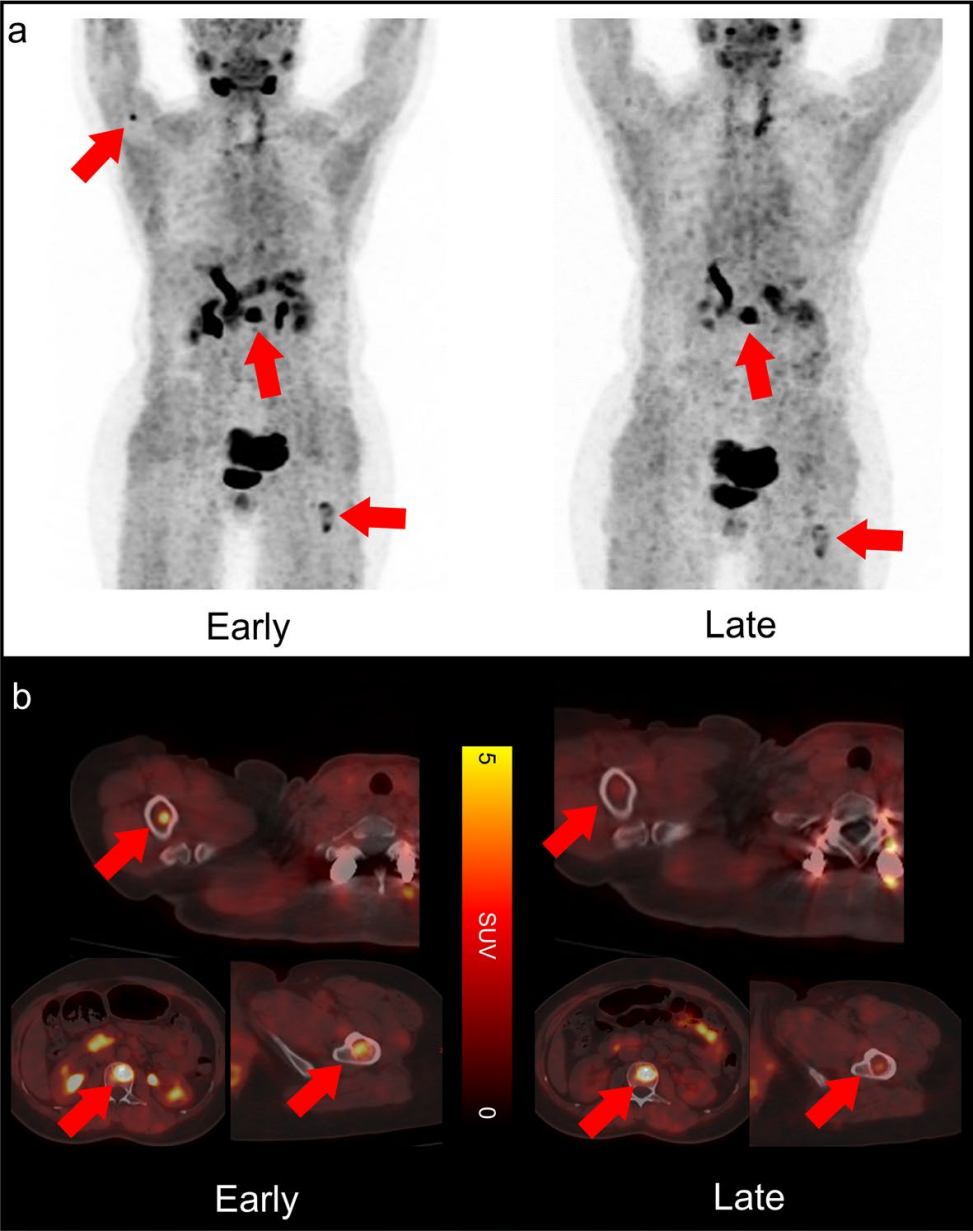
A 49-year-old woman with multiple bone metastasis of differentiated thyroid cancer. **a** Comparison of maximum-intensity projection images between early and late [^18^F]FAPI-42 PET (lesions indicated by arrows). **b** PET/CT images on early and late [^18^F]FAPI-42 PET (arrows indicate metastatic lesions). Early [^18^F]FAPI-42 PET detected more metastatic bone lesions than late [^18^F]FAPI-42 (SUV_max_ of humerus lesion is 4.9 and 2.4, respectively; SUV_max_ of vertebrae is 7.6 and 6.4, respectively; SUV_max_ of femur is 4.8 and 4.1)

**Fig. 4:**
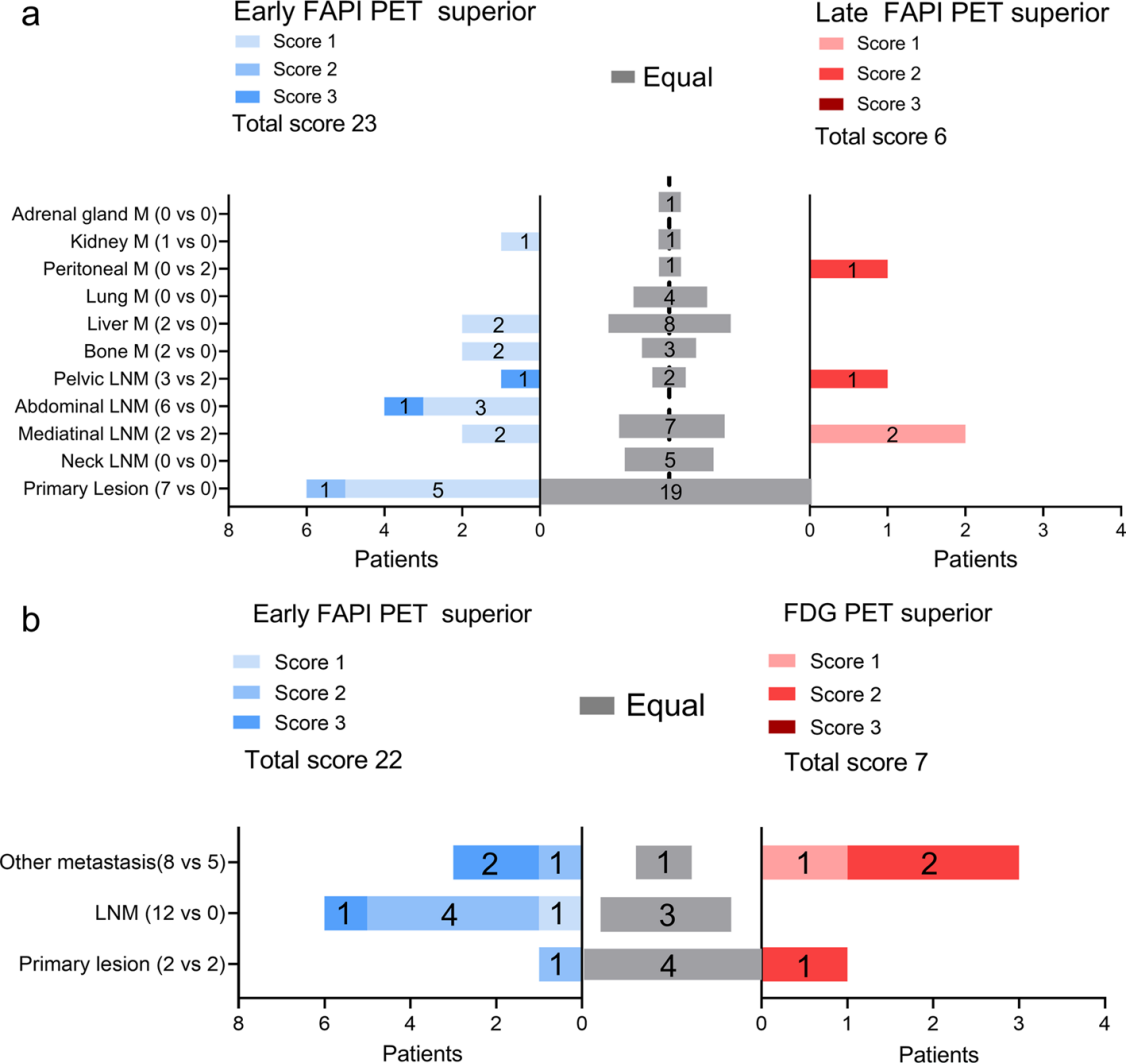
Comparison of visual score between early and late [^18^F]FAPI-42 PET in primary lesions, LN metastasis, and other metastasis (**a**) and visual score of lesions on early [^18^F]FAPI-42 PET compared with 2-[^18^F]FDG PET (**b**). LNM = Lymph node metastasis; M = Metastasis

In total, 194 lesions were detected in 60 patients. Lesions were rated as primary or recurrent lesions (29/194, 19.8%), lymph node metastasis (100/194, 21.5%), or other metastasis (65/194, 5.8%). Representative images were shown in Fig. 2. One of 194 (0.05%) of lesions were seen in early timepoint imaging but not in late timepoint imaging. That is a patient with differentiated thyroid cancer, referred for a metastatic bone assessment following a total thyroidectomy and 131-iodine ablation therapy (Fig. 3). For this case the disease stage did not change, as other lesions of the same region were visible. No additional lesions were observed on late imaging. For visual scoring system, there was a higher visual score was observed on early imaging (23 vs. 6, Fig. 4a).

**Fig. 5:**
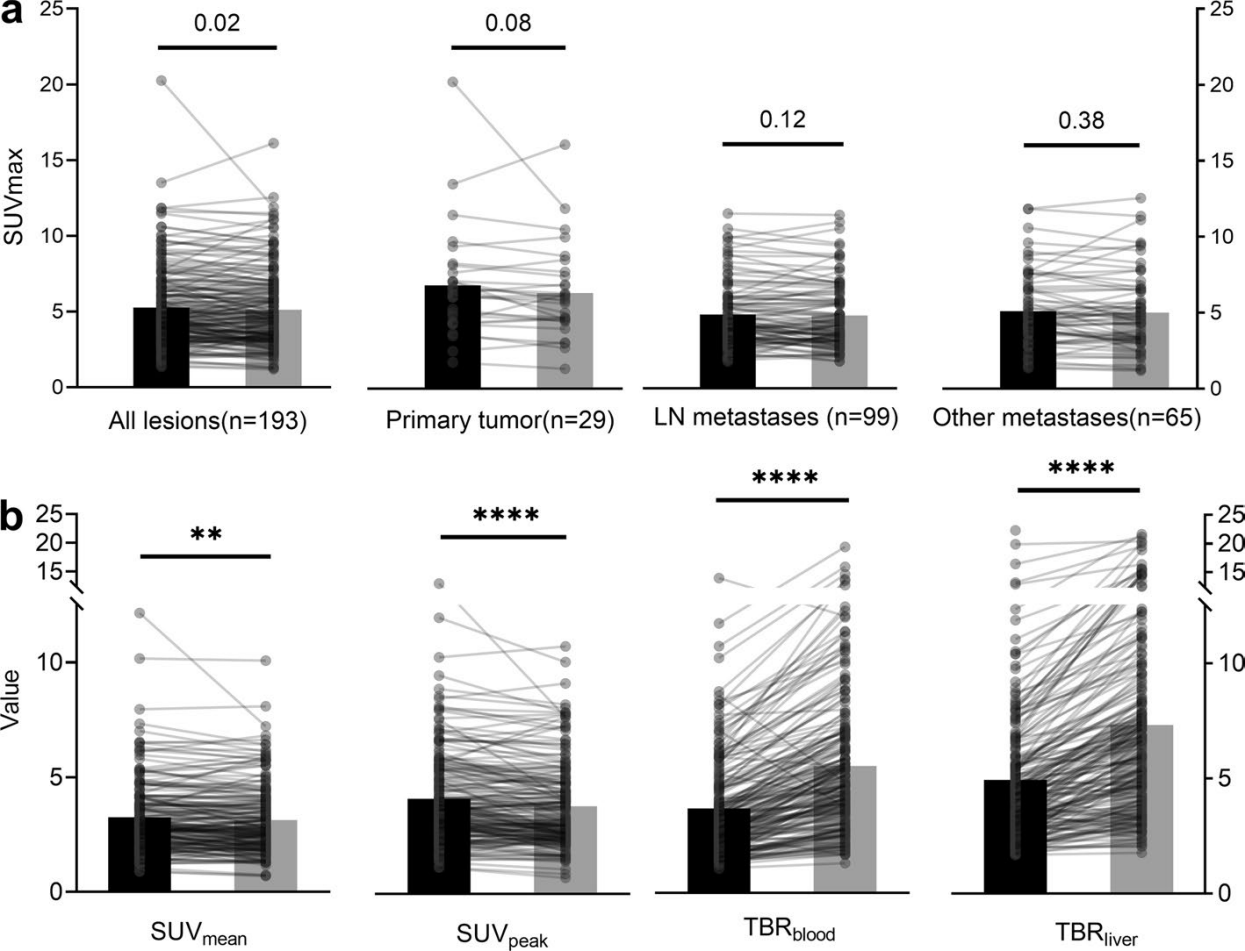
Comparison of uptake on [^18^F]FAPI-42 PET between early and late [^18^F]FAPI-42 PET: SUV_max_ in all lesions, primary lesions, LN metastases, and other metastases (**a**); SUV_mean_, SUV_peak_, TBR_blood_ and TBR_liver_ on [^18^F]FAPI-42 PET between early and late timepoint in all lesions (**b**). LN = Lymph nodes; SUV_max_ = Maximum standardized uptake value; SUV_mean_ = Mean standardized uptake value; SUV_peak_ = Peak standardized uptake value; TBR = Tumor-to-background ratio. Vertical bar shows the mean

Overall, the uptake of all lesions statistically decreased over time (median SUV_max_: 4.6 vs. 4.6; SUV_mean_: 2.9 vs. 2.8; SUV_peak_: 3.7 vs. 3.2, all *p* < 0.05; Fig. 4a). However, for individual anatomical regions, including primary or recurring lesions (median SUV_max_: 6.3 vs. 5.9, *p* = 0.08), lymph nodes (median SUV_max_: 4.3 vs. 4.2, *p* = 0.12), and distant metastases (median SUV_max_: 4.6 vs. 4.6, *p* = 0.38), no statistically significant differences in SUV_max_ between the early and late scan were observed (Fig. 5a). Furthermore, the SUV_mean_ and SUV_peak_ of all lesions were evaluated based on their anatomical locations (Fig. 5b, Additional file 1: Fig. S1). While the [^18^F]FAPI-42 activity in lesions showed higher uptake in the early scan compared to the late scan, the TBR increased over time due to a significant decrease in background activity from the early to late timepoint. Consequently, statistical analysis revealed a significant decrease in the median TBR _blood_ and TBR _liver_ across all lesions (median 3.2 vs. 4.8, 4.6 vs. 7.7; all *p* < 0.001, respectively, Fig. 5), irrespective of their specific anatomical locations (Additional file 1: Fig. S1).

### Comparative analysis of early [^18^F]FAPI-42 and 2-[^18^F]FDG PET/CT

**Fig. 6:**
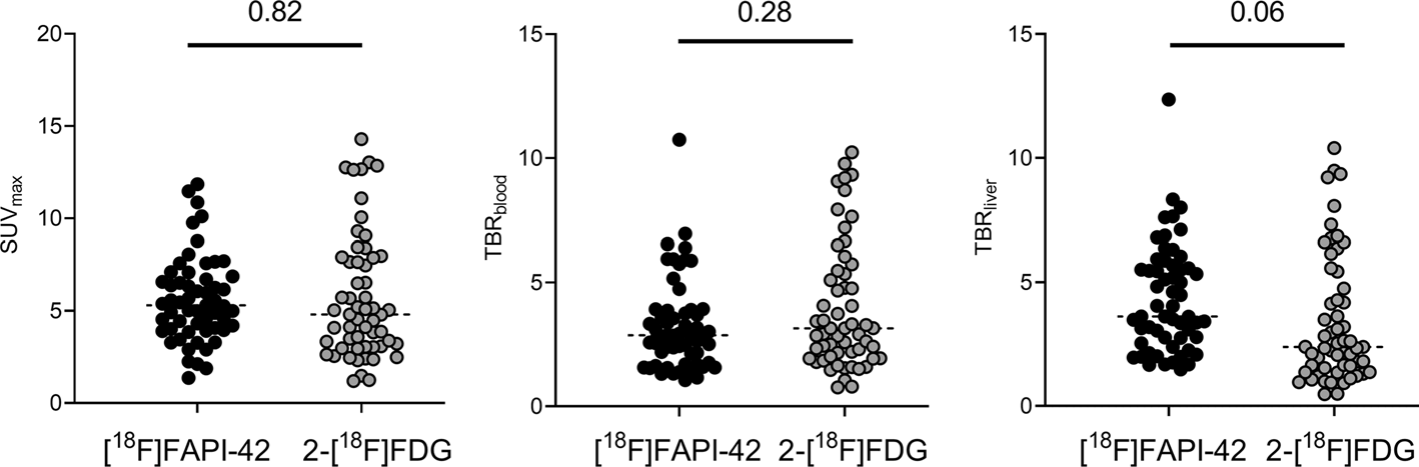
Quantitative comparisons (left, SUV_max_; middle, TBR_blood_; right, TBR_liver_) between early [^18^F]FAPI-42 and 2-[^18^F]FDG in all lesions; SUV_max_ = Maximum standardized uptake value; TBR = Tumor-to-background ratio

**Fig. 7:**
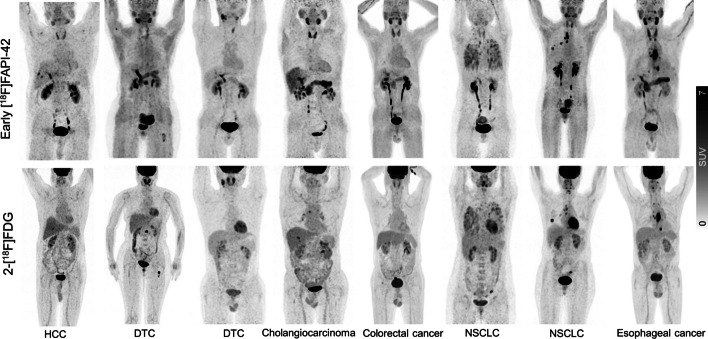
Maximum-intensity-projection images of early [^18^F]FAPI-42 and 2-[^18^F]FDG PET/CT in patients with different types of cancer. HCC = Hepatocellular carcinoma; DTC = Differentiated thyroid carcinoma; NSCLC = Non-small cell lung cancer

Table 2 presents the lesions between early [^18^F]FAPI-42 and 2-[^18^F]FDG in eleven patients. On the early [^18^F]FAPI-42 PET/CT, 57 malignant lesions were identified, in contrast to the 51 lesions spotted on 2-[^18^F]FDG PET/CT. Collectively, the visual scores for these patients were elevated in the early [^18^F]FAPI-42 PET/CT compared to 2-[^18^F]FDG PET/CT (22 vs. 7; Fig. 4b). In a quantitative assessment, the SUV_max_ for lesions was higher in the early [^18^F]FAPI-42 PET/CT than in 2-[^18^F]FDG PET/CT, though the difference wasn't statistically significant (median SUV_max_, 5.3 vs. 4.8, *p* = 0.818; Fig. 5). Similarly, there was no significant difference in the TBR_blood_ ratio for lesions between the two scans (median TBR_blood_ 2.9 vs. 3.1, *p* = 0.282; Fig. 6). Yet, when using liver radioactivity as a reference, the TBR_liver_ exhibited greater uptake in the early [^18^F]FAPI-42 PET/CT than in 2-[^18^F]FDG PET/CT (median TBR_liver_ 3.6 vs. 2.4, *p* = 0.057; Fig. 5). Representative PET images are presented in Fig. 7.

**Table 2:** Head-to-Head Comparison of SUV_max_ between Early [^18^F]FAPI-42 PET and 2-[^18^F]FDG PET

Caner type	State	Early [^18^F]FAPI-42 PET/CT					2-[^18^F]FDG PET/CT				
		Primary lesion	LN metastasis(1)	LN metastasis(2)	Other metastasis	Location	Primary lesion	LN metastasis(1)	LN metastasis(2)	Other metastasis	Location
Breast cancer	Recurrence				2.3	Bone				2.5	Bone
Colorectal cancer	Before surgery	7.1	6.1	6.2*	6.3, 3.8	Liver	14.3	3.2	4.1	12.6, 5.7	Liver
Colorectal cancer	Before surgery	6.3					7.9				
Cholangiocarcinoma	Before treatment	7.7			6.5, 5.7	Kidney	5.7			2.9, 3.0	Kidney
Esophagus cancer	Before surgery	11.5	7.6	6.8*			12.6	5.2	7.8		
Liver cancer	Recurrence	7.1					6.5				
NSCLC	Before Treatment	5.3	4.6	4.4*			5.1	2.4	2.5		
NSCLC	Before surgery	5	10.9	10.1*			7.6	5.1	5		
Prostate cancer	Before surgery	7.7					4.8				
Thyroid cancer	SIR^+^				7.6	Bone				8.4	Bone
Thyroid cancer	SIR^+^		8.8					7.9			

*Number of detected LN metastases is more than 3, and 2 major sites are shown here

Patient with multifocal pulmonary lesions, and the lesion with the largest diameter was shown here

^+^Follow-up examination indicate structural incomplete disease after thyroidectomy and radioactive iodine treatment

*LN* Lymph node; *NSCLC* Non-small cell lung cancer; *SIR* Structural incomplete disease

## Discussion

In this study, we compared the biodistribution, tracer retention, lesion detection, and uptake of early and late [^18^F]FAPI-42 PET. Both time points showed equal lesion detection; however, there was less tracer retention in the biliary system for the early time point. The uptake of normal organs and lesions was significantly higher for early PET acquisition, but it did not have a relevant impact on lesion detection or tumor staging due to the higher TBR for late acquisition. Furthermore, in some patients, early [^18^F]FAPI-42 PET demonstrated higher lesion detection and TBR compared to 2-[^18^F]FDG.

Hu et al. previously reported a rapid decrease in radioactivity in normal organs and satisfactory retention in tumors using [^18^F]FAPI-42 PET/CT with dynamic acquisition (Hu et al. 2022). Consistent with their findings, our study observed a similar decline in radioactivity from early to late imaging in normal organs, with no significant difference observed in bone tissue (median SUV_mean_ 0.88 vs. 0.85; p = 0.218). In contrast to ^68^[Ga]Ga-FAPI, the lipophilicity of [^18^F]FAPI leads to substantial accumulation in the biliary tract system, potentially limiting the detection of gastrointestinal lesions. Fortunately, our research suggests that early [^18^F]FAPI-42 imaging may reduce tracer accumulation in the biliary system, mitigating this drawback. Although some published studies have suggested that modifying [^18^F]FAPI ([^18^F]FAPT) can reduce hepatobiliary physiological excretion of ^18^F-labeled FAPI tracers, high tracer accumulation in other organs were observed up to 120 min after injection (Huang et al. 2023). Presently, no available data demonstrate whether this substantial accumulation in these organs will impact the diagnostic performance of [^18^F]FAPT. In this context, early acquisition appears to be a feasible strategy for [^18^F]FAPI-42, which is the most frequently utilized ^18^F-labeled FAPI tracer, as it helps mitigate the impact of excretion.

In a study by Ferdinandus et al., a comparison was conducted between early imaging (approximately 10 min post-injection) and late imaging (approximately 60 min post-injection) using [^68^ Ga]Ga-FAPI-46. They reported an equivalent detection rate between these two time points. Interestingly, they observed two lesions that were only visible in the early time point, although this did not alter the staging for the patients (Ferdinandus et al. 2021). They investigated three different time points for imaging with [^68^ Ga]Ga-FAPI-46 in various types of cancers (10 min, 60 min, and 180 min post-injection). Surprisingly, they found a similar detection rate among the three time points (Naeimi et al. 2023). Consistent with these earlier findings, our study also revealed an equal detection rate between the early and late timepoints for [^18^F]FAPI-42. This emphasizes the advantages of early time point acquisition for [^18^F]FAPI-42, as it maintains an equal detection rate while streamlining workflow and reducing patient waiting times.

Regarding lesion uptake, Ferdinandus et al. demonstrated that the mean SUVmax of lesions reached its peak at 10 min (8.2) and exhibited a slight decline at 1 h (8.15) and 3 h (7.6) after tracer administration (Naeimi et al. 2023). This observation is consistent with earlier reports, which indicated that lesion uptake reaches its peak around 20–30 min, followed by a gradual decrease until the 120-min mark. Our own data substantiate these findings, confirming the decline in lesion uptake over time. Moreover, in conjunction with the elevated lesion uptake, we noted a higher visual score for lesions in the early [^18^F]FAPI-42 images (23 vs. 6). Crucially, this outcome remained consistent and was not affected by a substantial increase in TBR for lesions over time. This observation can be valuable for aiding physicians in the interpretation of lesions on early [^18^F]FAPI-42 images. Notably, there was a significant discrepancy where one lesion was missed in late time-point imaging but detected in early timepoint imaging. We speculate that this inconsistency is linked to the decline in lesion uptake over time. Based on these findings, we can infer that a short waiting period after [^18^F]FAPI-42 administration suffices for effective lesion detection. Theoretically, a longer delay image of [^18^F]FAPI-42 (up to 120 min or more) may not be recommended, because prolonged delayed time does not yield higher tumor uptake. Therefore, even in cases where certain patients may require dual-time imaging with [^18^F]FAPI-42 to differentiate the nature of lesions, utilizing the early and late timepoints may be sufficient.

Despite the relatively complex preparation requirements, which include fasting, maintaining appropriate blood sugar levels, and extended waiting times, 2-[^18^F]FDG continues to serve as a well-established late for diagnostic imaging in various cancer types and as a guide for treatment planning. Consistent with existing literature, our research has demonstrated that early [^18^F]FAPI-42 detected more postive lesions than that of 2-[^18^F]FDG PET/CT (Yang et al. 2023; Wu et al. 2022; Lyu et al. 2023). Although there were no significant differences in SUV_max_ values for lesions between early [^18^F]FAPI-42 and 2-[^18^F]FDG, early [^18^F]FAPI-42 yielded higher visual scores for lesions, potentially attributed to the elevated TBR of lesions in early [^18^F]FAPI-42 (Lyu et al. 2023). However, despite the promising detection capabilities of [^18^F]FAPI-42, solely relying on imaging the tumor microenvironment with [^18^F]FAPI-42 may not be universally recommended or consistently adequate for the diagnostic assessment of malignant diseases, owing to inherent tumor heterogeneity. Given the distinct mechanisms of action and tracer retention, 2-[^18^F]FDG and [^18^F]FAPI-42 PET/CT may serve as complementary tools, capturing different aspects of tumor biology. Our results suggest that early [^18^F]FAPI-42 can serve as a valuable complementary tool alongside 2-[^18^F]FDG. This approach capitalizes on high lesion uptake and clinical feasibility, potentially leading to increased scan volumes, streamlined workflows, and reduced spatial requirements in the nuclear medicine department.

Our study has several limitations that should be considered. Firstly, one limitation stems from the absence of histological confirmation of the lesions. We did not include accuracy assessment as an endpoint, and formal lesion validation was not part of this analysis. This limitation could potentially result in the misclassification of lesions. Secondly, due to the limited number of patients underwent paired 2-[^18^F]FDG PET/CT in the study, reliable comparisons among different tumor types were not possible. Therefore, any conclusions drawn from our data comparing early [^18^F]FAPI-42 and 2-[^18^F]FDG should be made with caution.

## Conclusion

In conclusion, when compared to the late approach, early [^18^F]FAPI-42 PET acquisition yielded comparable lesion detection rates but exhibited higher lesion uptake and visual scores at an early timepoint. In a subgroup analysis, early [^18^F]FAPI-42 depicted more positive lesions with higher TBR than that of 2-[^18^F]FDG PET/CT. Thus, for increasing scan volumes and streamline workflows, acquisition at early timepoint could be a feasible protocol of [^18^F]FAPI-42 PET/CT.

### Supplementary Information

**Additional file 1. Table S1. **The biodistribution of ^18^F-FAPI-42 between early and standard scanTABLE 1. The biodistribution of ^18^F-FAPI-42 between early and standard scan. **Fig. S1.** Comparison of uptake on ^18^F-FAPI-42 PET between early and standard ^18^F-FAPI-42 PET.

## Data Availability

The datasets generated during and/or analysed during the current study are available from the corresponding author on reasonable request.
