# Supplementary material for: Comparative analysis of two timepoints on [18F]FAPI-42 PET/CT in various cancers
Source: Eur J Hybrid Imaging. 2023 Dec 11;7:27. doi: 10.1186/s41824-023-00186-1 (PMC10710973; doi:10.1186/s41824-023-00186-1)

**Supplementary materials**

**Visual scoring system**

If lesions detected by early FAPI PET showed an area, count, or tracer uptake that was: 1–3 times greater than normal FAPI PET, they scored 1;3–5 times greater, they scored 2; More than 5 times greater, they scored 3. Conversely, if lesions detected by normal FAPI PET had: 1–3 times the area, count, or tracer uptake of early FAPI PET, they scored 1; 3–5 times more, they scored 2; More than 5 times more, they scored 3. Any other scenarios resulted in a score of 0. The same criteria were applied when comparing early FAPI PET to FDG PET.

TABLE 1. The biodistribution of ^18^F-FAPI-42 between early and standard scan

|  | Early timepoint | Late timepoint | *p* value |
| --- | --- | --- | --- |
| Brain | 0.11(0.08,0.19) | 0.08(0.05,0.15) | <0.001 |
| Right parotid gland | 1.84(1.57, 2.67) | 1.12(0.95,1.53) | <0.001 |
| Left parotid gland | 1.93(1.42, 2.71) | 1.16(0.90,1.44） | <0.001 |
| Right salivary | 2.89(2.46,4.06) | 1.78(1.47, 2.31) | <0.001 |
| Left salivary | 2.96(2.38, 3.89) | 1.79(1.48,2.42) | <0.001 |
| Right thyroid | 2.53(1.82,3.24) | 1.44(1.15,1.75) | <0.001 |
| Left thyroid | 2.37(1.74,3.27) | 1.41(1.16,1.84) | <0.001 |
| Blood pool | 1.46(1.22,1.93) | 0.92(0.79,1.16) | <0.001 |
| Lung | 0.49(0.42,0.61) | 0.36(0.29,0.44) | <0.001 |
| Liver | 1.11(0.93,1.44) | 0.67(0.58,0.61) | <0.001 |
| Spleen | 1.05(0.88,1.34) | 0.64(0.56,0.77) | <0.001 |
| Pancreas | 2.31(1.88,3.22) | 1.33(0.93,1.67) | <0.001 |
| Kidney | 2.15(1.61,2.72) | 1.54(1.05,1.88) | <0.001 |
| Muscle | 1.08(0.78,1.33) | 0.98(0.72,1.21) | <0.001 |
| Bone | 0.88(0.63,1.17) | 0.85(0.60,1.08) | 0.218 |

Fig. 1 Comparison of uptake on ^18^F-FAPI-42 PET between early and standard ^18^F-FAPI-42 PET.


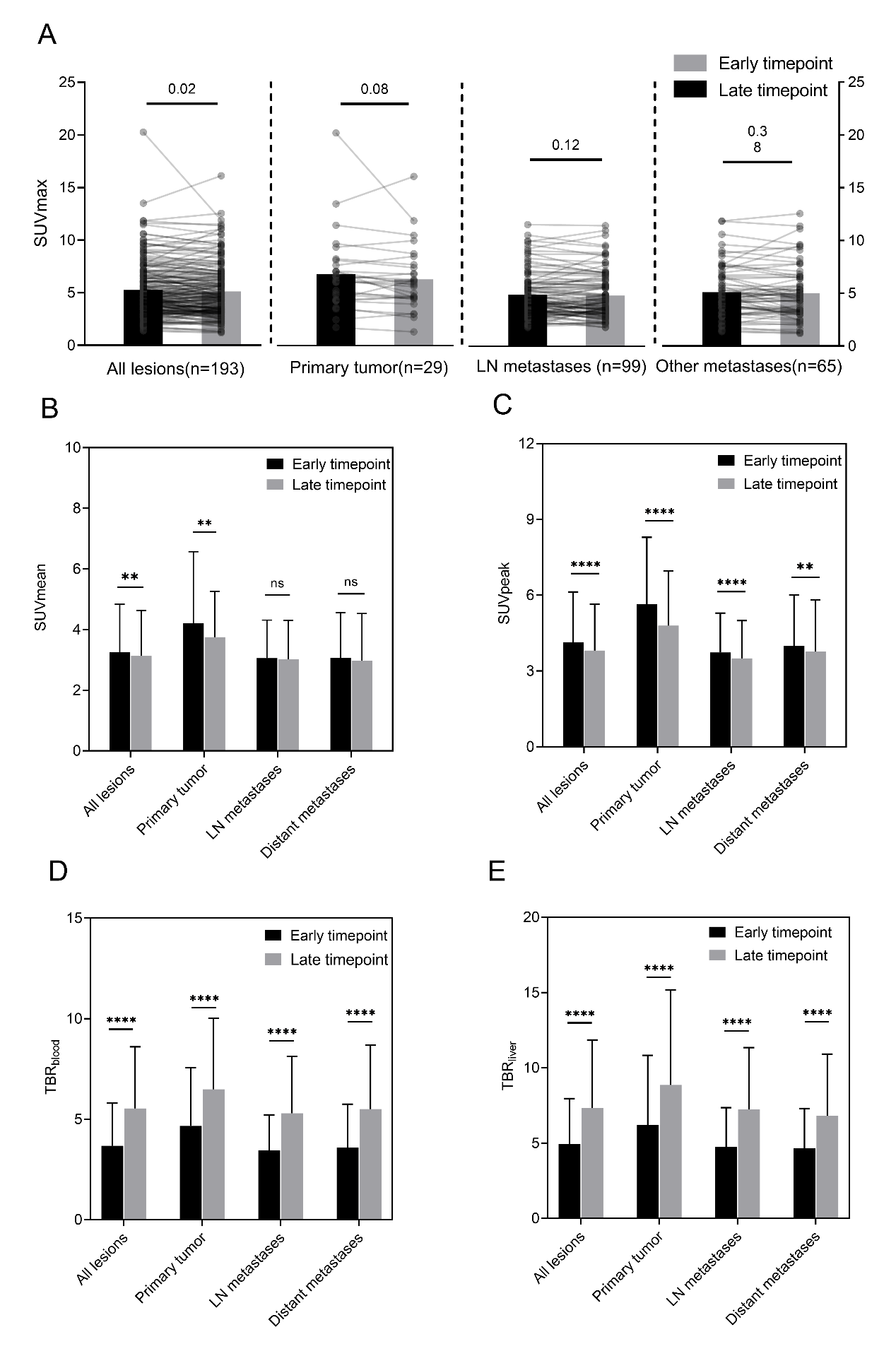

Supplement: Supplementary file 1 — Additional file 1. Table S1. The biodistribution of 18F-FAPI-42 between early and standard scanTABLE 1. The biodistribution of 18F-FAPI-42 between early and standard scan. Fig. S1. Comparison of uptake on 18F-FAPI-42 PET between early and standard 18F-FAPI-42 PET. [file 41824_2023_186_MOESM1_ESM.docx]
